# Enhancing Security and Flexibility in the Industrial Internet of Things: Blockchain-Based Data Sharing and Privacy Protection

**DOI:** 10.3390/s24031035

**Published:** 2024-02-05

**Authors:** Weiming Tong, Luyao Yang, Zhongwei Li, Xianji Jin, Liguo Tan

**Affiliations:** 1 Laboratory for Space Environment and Physical Sciences, Harbin Institute of Technology, Harbin 150001, China; tanliguo@hit.edu.cn; 2 School of Electrical Engineering and Automation, Harbin Institute of Technology, Harbin 150001, China; 22b306009@stu.hit.edu.cn (L.Y.); lzw@hit.edu.cn (Z.L.); mrking@hit.edu.cn (X.J.)

**Keywords:** blockchain, data sharing, attribute-based encryption, zero-knowledge proof

## Abstract

To address the complexities, inflexibility, and security concerns in traditional data sharing models of the Industrial Internet of Things (IIoT), we propose a blockchain-based data sharing and privacy protection (BBDSPP) scheme for IIoT. Initially, we characterize and assign values to attributes, and employ a weighted threshold secret sharing scheme to refine the data sharing approach. This enables flexible combinations of permissions, ensuring the adaptability of data sharing. Subsequently, based on non-interactive zero-knowledge proof technology, we design a lightweight identity proof protocol using attribute values. This protocol pre-verifies the identity of data accessors, ensuring that only legitimate terminal members can access data within the system, while also protecting the privacy of the members. Finally, we utilize the InterPlanetary File System (IPFS) to store encrypted shared resources, effectively addressing the issue of low storage efficiency in traditional blockchain systems. Theoretical analysis and testing of the computational overhead of our scheme demonstrate that, while ensuring performance, our scheme has the smallest total computational load compared to the other five schemes. Experimental results indicate that our scheme effectively addresses the shortcomings of existing solutions in areas such as identity authentication, privacy protection, and flexible combination of permissions, demonstrating a good performance and strong feasibility.

## 1. Introduction

With the rapid advancement of computing, communication, and artificial intelligence technologies, a plethora of traditional internet technologies have been integrated into the Industrial Internet of Things (IIoT), significantly enhancing the rate of data transmission and sharing [1,2]. The core operations in IIoT data transfer and sharing involve the interconnectivity of sensors, communication nodes, and control systems to gather, transmit, and analyze data. This data includes sensitive information about equipment status, production processes, and supply chains [3,4]. However, as the IIoT increasingly merges with traditional information technologies, it faces a growing number of cybersecurity challenges [5]. Due to limitations in resources and hardware, the IIoT cannot implement complex and precise security protections like traditional information systems. This makes it vulnerable to attacks during data transmission and sharing processes [6,7].

In the past year, there have been several reported incidents of data breaches involving the IIoT. For instance, the health management company Intellihartx confirmed that hackers stole the medical information and social security numbers of over half a million patients [8]. The MOVEit file transfer tool (v. 13.0.7, v. 13.1.5, v. 14.0.5, v. 14.1.6, v. 15.0.2) was attacked, leading to the leak of sensitive data from companies like wage service provider Zellis, British Airways, BBC, and Nova Scotia, which used the software [9]. The American pharmaceutical giant PharMerica disclosed that unknown actors accessed its system in March and extracted the personal data of 5.8 million individuals [10]. A ransomware hacker group named BlackCat threatened to leak 80 GB of confidential data they claimed to have stolen from Reddit servers in February [11]. According to a 2022 report by Waterfall Security Solutions, there were 57 cyber-attack incidents related to operational technology, impacting over 150 industrial operations [12]. This suggests an escalation in industrial network security challenges, with projections indicating that upwards of 15,000 industrial sites may face operational cessation due to cyber incursions within the ensuing five-year period [13,14]. In the event of a sustained increase in malicious attacks targeting the industrial internet, the fragility of such networks is expected to be severely threatened, potentially impacting the regular operations of the IIoT systems. [15,16]. Hence, there is an urgent need to develop a convenient, flexible, and secure data sharing solution for the IIoT.

Blockchain technology, as a decentralized and secure distributed ledger system, offers a novel approach to resolving the challenges of secure data sharing within the IIoT. It provides a decentralized platform for data management and sharing, ensuring data security and privacy [17,18]. The distributed and immutable nature of blockchain technology complicates hacking attempts and data tampering. Additionally, blockchain’s consensus mechanism and data auditing capabilities increase the trustworthiness and transparency of data, allowing stakeholders to share and use reliable data [19,20]. However, nodes in a blockchain are required to store and process all transaction data, which significantly increases the blockchain’s storage and computational requirements as data volume grows [21]. The inherent public and transparent nature of blockchain, where all transactions and data are accessible to participating nodes, poses challenges to protecting the privacy of the IIoT users and members [22]. Although blockchain technology opens new possibilities for addressing IIoT data security sharing issues, due to these factors its direct application to the IIoT still faces some difficulties.

Addressing the outlined issues, this work combines weighted threshold secret sharing, zero-knowledge proof, and attribute-based encryption technologies to propose a blockchain-based data sharing and privacy protection (BBDSPP) scheme for the IIoT. This scheme utilizes the weighted threshold secret sharing method to characterize and assign values to attributes, achieving a flexible combination of permissions. This allows terminal members to access system data securely and flexibly. The scheme also incorporates a non-interactive zero-knowledge proof protocol to pre-authenticate data accessors, preventing unauthorized access and data leakage. Moreover, it employs the interplanetary file system (IPFS) for distributed storage of encrypted data, reducing the storage pressure on the blockchain.

### 1.1. Main Contributions

The main contributions of this paper can be summarized as follows:

(1) Design of Data sharing Architecture: We have summarized the advantages and disadvantages of various existing data sharing schemes and analyzed the security risks associated with data sharing in the IIoT. We designed a data sharing system architecture suitable for the IIoT. This architecture comprehensively considers the flexibility, security, and scalability of data sharing. Based on blockchain technology, it achieves efficient, secure, and transparent data sharing in a decentralized manner, ensuring the confidentiality and integrity of critical data and providing a solution to the security needs of the IIoT;
(2) Improvement of the Data Sharing Scheme: We assign values to attributes based on their characteristics and use a weighted threshold secret sharing scheme to improve the data sharing approach, creating a data sharing scheme with freely combinable permissions. This scheme allows terminal members to freely select the attributes for decryption. Access to specific data is granted once the attribute values meet the preset access threshold. Not only does this scheme ensure the flexibility of data sharing, but it also enhances the rigor of access control, achieving fine-grained access control in the system;
(3) Implementation of Privacy Protection: Based on non-interactive zero-knowledge proof technology, we have designed a lightweight identity verification protocol to pre-validate the identity of data accessors. This ensures that only authorized terminal members can access system data, preventing identity impersonation by unauthorized members and illegal access to sensitive data. Consequently, this secures the privacy of terminal members and the safety of data while also reducing the additional computational overhead caused by illegal access attempts;
(4) Distributed Data Storage: We store a substantial amount of data on the IPFS, and only the corresponding storage addresses are recorded on the blockchain. This storage method not only ensures the security and integrity of the data but also significantly enhances data retrieval efficiency and the scalability of the system. This approach addresses the issue of insufficient storage space in traditional blockchain applications;
(5) Performance Analysis and Validation: We conducted a thorough analysis of the proposed solution in terms of correctness and security, along with a comprehensive theoretical assessment of its functional characteristics and computational costs. Additionally, we validated the practical effectiveness of the solution through experimental design. The experimental results indicate that the scheme effectively overcomes the limitations found in existing data sharing approaches in critical areas such as identity authentication, privacy protection, flexible permission configuration, and off-chain storage, while also demonstrating superior performance and strong feasibility.

### 1.2. Organization

In Section 2, we describe the related work of this paper; in Section 3, we introduce the basic knowledge; in Section 4, we provide a detailed introduction to the BBDSPP scheme; in Section 5, we analyze the correctness and security of BBDSPP; in Section 6, we further analyze the performance of BBDSPP; and in Section 7, we summarize the content of the entire paper.

## 2. Related Work

In recent years, blockchain-based data sharing and privacy protection schemes for the IIoT have become a widely researched topic of international interest. Numerous scholars have delved into the exploration of data sharing and privacy protection [23], yielding a series of significant research achievements in these fields. Key developments in this area are outlined in Table 1 and Table 2.

Based on the analysis of the aforementioned literature, numerous scholars have made significant contributions to research on blockchain technology’s application in data sharing and privacy protection in the IIoT. These studies lay a solid foundation for the research approach of this paper. However, there are still several challenges in these research outcomes, including the complexity of data sharing algorithms, lack of flexibility, insufficient security, and relatively high computational costs. Moreover, these studies have not been closely integrated with the structural characteristics of the industrial internet, limiting their application in complex IIoT environments. Building upon current research, several critical issues need to be addressed, as analyzed below:

(1) Achieving efficient data transmission and sharing without sacrificing algorithm performance;
(2) Effectively managing and verifying the identities of a large number of devices and handling the vast amount of data generated;
(3) Ensuring the integrity and security of data during its collection, transmission, and storage in environments with multiple stakeholders.

Therefore, it is imperative to investigate a data sharing and privacy protection scheme suitable for the IIoT which enables secure and efficient data sharing among terminal members while safeguarding their privacy information. For this purpose, we propose the BBDSPP scheme, which integrates cutting-edge technologies such as weighted threshold secret sharing, zero-knowledge proof, and attribute-based encryption. In this scheme, we assign characteristic values to attributes and optimize the data sharing process, thus implementing a flexible access control mechanism with combinational permissions. Subsequently, utilizing non-interactive zero-knowledge proof protocols, we pre-authenticate the identities of data accessors, effectively preventing unauthorized access. Moreover, we employ the IPFS distributed storage system to alleviate the storage burden on the blockchain, enhancing storage efficiency and system scalability. This approach provides a secure and trustworthy environment for data circulation in the IIoT.

## 3. Basic Concepts

In this section, we primarily introduce the fundamental mathematical concepts utilized in the BBDSPP scheme, including the three basic properties of bilinear mapping, the definition and three fundamental properties of weighted threshold secret sharing, and the basic principles of zero-knowledge proofs. The zero-knowledge proof protocols encompass both interactive and non-interactive types. In the authentication phase of the BBDSPP scheme, we employ a non-interactive zero-knowledge proof protocol. To facilitate a better understanding for the readers, in this section, we introduce and compare the principles of both interactive and non-interactive zero-knowledge proof protocols. In this scheme, we characterize and assign values to attributes to optimize the data sharing process, achieving a flexible access control mechanism with combinational permission settings.

### 3.1. Bilinear Mapping

Let $G_{1}$, $G_{2}$, and $G_{T}$ be cyclic groups of prime order p. A bilinear pairing is a map $e:G_{1}\times G_{2}\to G_{T}$, where $G_{T}$ is also a cyclic group of the same order, satisfying the following properties [36]:

(1) Bilinearity: For all $u\in G_{1}$, $v\in G_{2}$, and $a,b\in Z_{q}^{*}$, the map satisfies $e(u^{a},v^{b})=e{\left(u,v\right)}^{ab}$;
(2) Non-degeneracy: For all $u\in G_{1}$ and $v\in G_{2}$, the map does not send every pair to the identity element of $G_{T}$, that is e(u,v)≠1;
(3) Computability: For all $u\in G_{1}$ and $v\in G_{2}$, there exists an efficient algorithm to compute e(u,v).

Additionally, the bilinear map is required to have the following uniqueness property: for any $u_{1},u_{2}\in G_{1}$, and $v\in G_{2}$, if $e(u_{1},v)=e(u_{2},v)$ then $u_{1}=u_{2}$.

### 3.2. Weighted Threshold Secret Sharing

Weighted threshold secret sharing (WTSS) is an extension of the threshold secret sharing scheme originally proposed by Shamir in 1979. In traditional threshold secret sharing schemes, each participant is considered to have an equal contribution to the reconstruction of the secret. However, in practical applications, the status and role of participants often differ. To address this issue, researchers have introduced the WTSS mechanism. The core concept of this mechanism is to distribute a secret among a group of participants, where each participant is assigned a weight [37]. This weight reflects the participant’s importance or level of trust within the group.

Definition: In a WTSS scheme, a secret s is divided into n shares $s_{1},s_{2},\ldots ,s_{n}$, and distributed to n participants. Each participant $P_{i}$ is assigned a weight $w_{i}$. The secret s can be reconstructed only when the sum of the weights of the participants who combine their shares is greater than or equal to a predefined threshold W.

Properties:

(1) Flexibility: WTSS allows for a flexible and hierarchical structure of trust among participants by assigning different weights to each participant;
(2) Security: The secret cannot be reconstructed unless the weighted shares sum up to at least the threshold, providing security against partial compromise;
(3) Robustness: WTSS schemes are robust against the failure of some participants to provide their shares, as long as the threshold can still be reached with the available shares.

In summary, WTSS provides a means for securely distributing a secret in a manner that reflects the hierarchy and trust levels within a group, ensuring that only a weighted combination of participants can reconstruct the secret [38].

### 3.3. Zero-Knowledge Proofs

Zero-knowledge proofs can be categorized into interactive and non-interactive types. Interactive zero-knowledge proofs involve multiple rounds of information exchange between the prover and the verifier. In this method, the prover begins with a preliminary proof. Subsequently, the verifier poses a series of random challenges based on this proof, to which the prover must respond appropriately. This process is repeated until the verifier is convinced of the proof’s validity. If the statement to be proven is true, an honest prover can convince the verifier of its truth without revealing any additional information. The Schnorr protocol is a classic example within this category of proofs, used to demonstrate knowledge of a discrete logarithm without disclosing its value [39].

**Figure 1:**
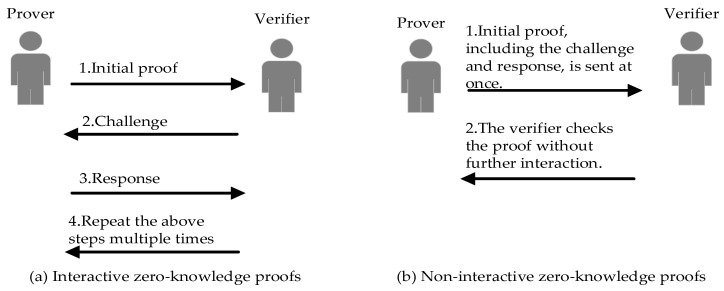
Comparison between Interactive zero-knowledge proofs and Non-interactive zero-knowledge proofs.

Non-interactive zero-knowledge proofs, on the other hand, involve a one-way transmission of proof from the prover to the verifier without further interaction. The advantage of this method is that the prover can pre-generate a proof and reuse it across multiple scenarios, eliminating the need for ongoing interaction with the verifier. To achieve non-interactivity, techniques such as a “common reference string” or cryptographic tricks like the Fiat–Shamir heuristic are often employed to convert interactive protocols into a non-interactive format. Zero-knowledge succinct non-interactive arguments of knowledge (zk-SNARKs) are a widely-used form of zero-knowledge proofs, particularly applied within blockchain technology and extensively used on platforms like Ethereum [40]. A comparison between interactive and non-interactive zero-knowledge proofs is illustrated in Figure 1.

## 4. Data Security Sharing Schemes

In this section, we provide a detailed introduction to the core components of the BBDSPP scheme, which include the participating entities, the system model, and the execution process. Firstly, we systematically elucidate the functions and responsibilities of the participating entities to ensure that readers have a clear comprehension of each entity’s role within the scheme. Following this, we present the framework of the system model, aiming to furnish readers with a comprehensive understanding of the system’s operational mechanisms. Lastly, we describe the specific steps involved in the implementation of the scheme, covering the application of key technologies and the execution details of each phase. The detailed introduction provided in this chapter aims to offer readers an in-depth and systematic understanding of the BBDSPP scheme.

### 4.1. Scheme Entities

In this paper, we propose the BBDSPP scheme, which is centered around the core idea of assigning values to attributes based on their characteristics. This is achieved through the utilization of a weighted threshold secret sharing scheme which improves the data sharing process, facilitating a flexible and secure data sharing access control mechanism that allows IIoT end members access to data in the system both flexibly and securely. Moreover, the scheme employs a non-interactive zero-knowledge proof protocol for pre-authenticating the identities of data accessors, preventing the impersonation by unauthorized members, theft of sensitive data, and unnecessary computational overhead. Additionally, the scheme leverages the IPFS distributed storage system to store encrypted shared resources, recording only the storage addresses on the blockchain, significantly alleviating the blockchain’s storage burden.

In this scheme, entities involved in data sharing include: certificate authority (CA), attribute authority (AA), regulatory node (RN), end members, and the interplanetary file system (IPFS). The specific roles are described as follows:

(1) Certificate Authority (CA): As a trusted third party, the CA’s main function is to generate the system’s public parameters and the public keys for end members. It also generates signatures for end members based on the zero-knowledge proof protocol, which are used for identity authentication during data access;
(2) Attribute Authority (AA): The AA is responsible for assigning attributes to end members, associating these attributes with the unique identity identifier $id_{i}$ of the end members, and registering them. Additionally, the AA categorizes attributes within the system;
(3) Regulatory Node (RN): The RN, a set of pre-selected nodes within a consortium blockchain, is tasked with monitoring and recording a series of data transactions of end members. It forms a public ledger through a secure consensus algorithm;
(4) End Members: End members are smart terminal devices in the IIoT, including data owners (DOs) and data visitors (DVs). DOs are end members that offer data for sharing, primarily focusing on customizing data access policies, encrypting data, and publishing data. DVs are end members that request access to data, mainly submitting data access applications, downloading ciphertext, and decrypting it. The roles of DOs and DVs are interchangeable. A DO can also act as a DV for data querying and access, and vice versa;
(5) Interplanetary File System (IPFS): As a distributed storage platform, IPFS securely stores encrypted shared resources uploaded by DO and returns storage addresses. These addresses serve as clues for DV to download shared resources.

The characters used in this scheme and their meanings are shown in Table 3.

### 4.2. System Model

The BBDSPP scheme’s system model primarily ensures the privacy protection and secure sharing of data information through aspects such as identity authentication, encrypted storage, and access control. The scheme comprises five parts: the initialization phase, registration phase, encryption phase, authentication phase, and decryption phase, and is executed by five participating entities: CA, AA, RN, end members, and IPFS.

**Figure 2:**
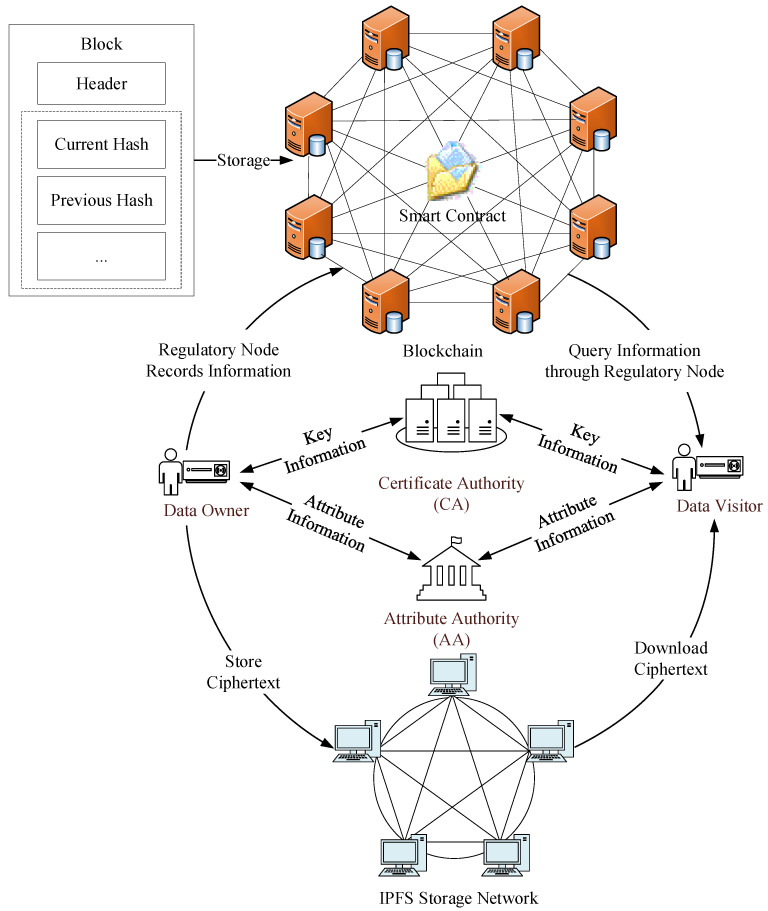
The BBDSPP scheme’s system model.

The system model of the BBDSPP scheme is depicted in Figure 2.

During the initialization phase, the CA generates public and private keys for end members based on their identity identifiers. The private key is securely stored and the public key is combined with other parameters to form the system’s public parameters. In the registration phase, the AA first assigns attributes to end members. Then, end members submit their attribute values and public keys to the CA. The CA authenticates end members using a non-interactive zero-knowledge proof protocol, which facilitates anonymous authentication during data access and protects the privacy of data requesters.

In the encryption phase, the DO uses a symmetric encryption algorithm to encrypt the information M to be shared and stores the encrypted ciphertext on the IPFS. The symmetric key is then encrypted using a ciphertext-policy attribute-based encryption method with weighted attributes, yielding the ciphertext and storage address. Subsequently, the RN records the information on the blockchain according to its storage structure and forms a public ledger through a secure consensus algorithm.

In the authentication phase, the DV first locates the storage address of the desired information on the IPFS in the blockchain and submits their zero-knowledge proof to the DO. After the DO verifies the identity of the data requester, they send the data storage address to the data requester.

In the decryption phase, the DV locates and downloads the ciphertext of the desired data from the addressed location. The data is then decrypted using a generated decryption key. Successful decryption and data access occurs only if the attribute weights held by the DV meet the threshold access value; otherwise, decryption is not possible.

**Figure 3:**
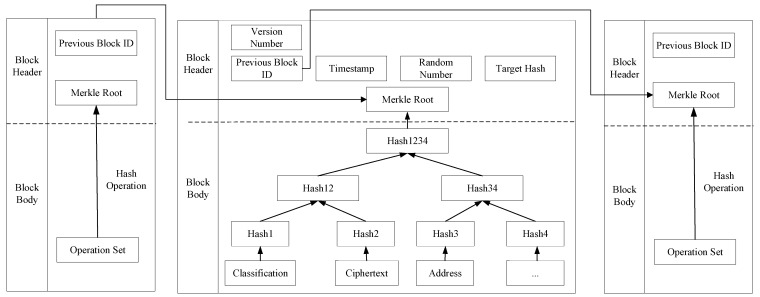
Blockchain data storage structure.

Blockchains use a chained structure to store and organize data where each block consists of a block header and a block body. The block header contains information such as the ID of the previous block, version number, and Merkle root, while the block body includes hash operations and transaction information. Blocks are connected to each other through hash pointers. The data storage structure of the blockchain is illustrated as shown in Figure 3.

In this scheme, end members effectively integrate and store the multi-dimensional information and related attributes of data in a blockchain, providing the DV with a more convenient and efficient method for data querying and retrieval. The data information in the blocks includes attribute classification (Classification), encrypted information of the symmetric key (Ciphertext), and storage address (Address), among others. In this scheme, “Classification” provides precise categorization information of accessible attributes, allowing the DV to quickly locate the required data based on their attributes. “Ciphertext” provides encrypted information of the symmetric key encrypted using attribute-based encryption. The DV can access the desired data only after decrypting to obtain the symmetric key. “Address” gives the storage location of the ciphertext in the IPFS, enabling the DV to find and download the ciphertext of the data they wish to access.

### 4.3. BBDSPP Scheme

Assuming that there are n terminal devices in the entire system, and these n terminal devices are regarded as n end members, then $U=\{u_{i}|i=1,2,\ldots ,n\}$ represents the set of these n end members. The corresponding set of identity identifiers is $ID=\{id_{i}|i=1,2,\ldots ,n\}$, where 1≤i≤n. Next, we define an ordered set of attributes as $A=\left\{a_{1},a_{2},\ldots ,a_{r}\right\}$, with corresponding attribute values $V=\left\{v_{1},v_{2},\ldots ,v_{r}\right\}$, and corresponding attribute weights $W=\left\{w_{1},w_{2},\ldots ,w_{r}\right\}$, where $r\in N^{*}$, indicating the total number of attributes. The BBDSPP scheme consists of five algorithms: initialization, registration, encryption, key generation, and decryption. Below are detailed explanations of the five steps.

(1) $Setup\left(id_{i}\right)\to \left\{Q_{U},pp\right\}$ Initialization Algorithm

This algorithm is carried out by the CA institution. It takes as input the identity identifier $id_{i}$ of the end members and outputs the public key $Q_{U}$ of the end member $u_{i}$ and the public parameters pp of the system. The specific process is as follows:

  i. Randomly select large prime numbers p and q, choose a bilinear group $G_{1}$ of prime order p, and a cyclic group $G_{2}$ of prime order q, with $g_{1}$ as the generator of $G_{1}$, and $g_{2}$ as the generator of $G_{2}$.
 ii. Define a bilinear pairing operation $e:G_{1}\times G_{1}\to G_{T}$.
iii. Choose three hash functions $H_{1}$ and $H_{2}$, where $H_{1}:{\left\{0,1\right\}}^{*}\to G_{1}$, and $H_{2}:{\left\{0,1\right\}}^{*}\to G_{2}$, and compute the public key of the end member $u_{i}$ as:
  (1) $$Q_{U}=H_{1}\left(id_{i}\right)$$
 iv. Select a random number $\alpha \in Z_{p}^{*}$, and compute the private key of the end member based on $Q_{U}$ as follows:
  (2) $$S_{U}=\alpha Q_{U}$$
  v. Then compute $u=e{\left(g_{1},g_{1}\right)}^{\alpha }$, hence the public parameters pp are:
  (3) $$pp=\left\{p,q,G_{1},g_{1},H_{1},H_{2},Q_{U},u\right\}$$

The process of the initialization algorithm is as follows (Algorithm 1):
**Algorithm 1:** $Setup\left(id_{i}\right)\to \left\{Q_{U},pp\right\}$// Params: identity of terminal member $id_{i}$ // Returns: public key $Q_{U}$ and public parameters pp function GeneratePublicKeyAndParams(char[] $id_{i}$) → {$Q_{U}$, pp} 01: // Step (i): Select large prime numbers and corresponding groups 02: p = selectLargePrime () 03: q = selectLargePrime () 04: $G_{1}$ = selectBilinearGroupOfOrder (p) 05: $G_{2}$ = selectCyclicGroupOfOrder (q) 06: $g_{1}$ = $G_{1}$.generator () // Generator of $G_{1}$ 07: $g_{2}$ = $G_{2}$.generator () // Generator of $G_{2}$ 08: // Step (ii): Define bilinear map 09: e = defineBilinearMap ($G_{1}$, $G_{1}$, $G_{T}$) 10: // Step (iii): Select hash functions 11: $H_{1}$ = hashFunction (${\left\{0,1\right\}}^{*}$, $G_{1}$) 12: $H_{2}$ = hashFunction (${\left\{0,1\right\}}^{*}$, $G_{2}$) 13: // Compute public key for terminal member $u_{i}$ 14: $Q_{U}=H_{1}\left(id_{i}\right)$ 15: // Step (iv): Select random number α and compute private key for terminal member $u_{i}$ 16: α = selectRandomFromZpStar (p) 17: $S_{U}=\alpha Q_{U}$ 18: // Step (v): Compute u and set public parameters 19: $u=e{\left(g_{1},g_{1}\right)}^{\alpha }$ 20: $pp=\left\{G_{1},g_{1},H_{1},H_{2},Q_{U},u\right\}$ 21: // Return public key and parameters 22: return {$Q_{U}$, pp} endfunction

(2) $Signup\left(V_{i},S_{U}\right)\to \left\{EV\right\}$ Registration Algorithm

The algorithm is jointly completed by the CA and the AA. The inputs to the algorithm are the attribute value $V_{i}$ and the public key $Q_{U}$. The outputs are the signature σ and the registration information table. The specific process is as follows:

The end member first submits their unique identity identifier to the AA. Upon receiving the application, the AA will assign attributes to the end member, including the name of the attribute, its value, and the corresponding weight value, and categorize these attributes before securely transmitting them to the end member. Let us assume that for the end member $u_{i}$, the corresponding unique identity identifier is $id_{i}$, the possessed attribute set is $A_{i}=\left\{a_{i_{1}},a_{i_{2}},\ldots ,a_{i_{r}}\right\}$, the corresponding attribute values are $V_{i}=\left\{v_{i_{1}},v_{i_{2}},\ldots ,v_{i_{r}}\right\}$, and the corresponding attribute weights are $W_{i}=\left\{w_{i_{1}},w_{i_{2}},\ldots ,w_{i_{r}}\right\}$, where $i,r\in N^{*}$, with i denoting the sequence number of the end member and r representing the number of attributes possessed by the i-th end member. Then, the AA registers the assigned attributes in association with the unique identity identifier $id_{i}$ of the end member. To prevent impersonation by unauthorized individuals, this process is only conducted once.

After receiving the attribute information, the end member sends the attribute information and the unique identity identifier $id_{i}$ to the CA. Upon receiving the information, the CA registers it and generates a zero-knowledge proof of the private key for the end member using a non-interactive zero-knowledge proof protocol, which serves as identity verification prior to the data access process. The detailed process is as follows:

  i. Compute T based on p and q from the public parameters:
  (4) T=p⋅q
 ii. Input the attribute values $V_{i}=\left\{v_{i_{1}},v_{i_{2}},\ldots ,v_{i_{r}}\right\}$ and the private key $S_{U}$, then compute the following formula:
  (5) $$F=|H_{2}\left(S_{U}\right)\left[V_{i}\right]{|}^{2}\;=|H_{2}\left(S_{U}\right)\left[v_{i_{1}},v_{i_{2}},\ldots ,v_{i_{r}}\right]{|}^{2}\;=|l_{1},l_{2},\ldots ,l_{r}{|}^{2}\;=\left\{l_{1}^{2},l_{2}^{2},\ldots ,l_{r}^{2}\right\}\;=\left\{f_{1},f_{2},\ldots ,f_{r}\right\}$$

Finally, the proof $EV=\left\{T,F\right\}$ is obtained and sent to the end member through a secure channel, along with the relevant parameters.

The process of the registration algorithm is as follows (Algorithm 2):
**Algorithm 2:** $Signup(V_{i},S_{U})\to \{EV\}$// Params: attribute values $V_{i}$; private key $S_{U}$ // Returns: proof EV function GenerateSignature(attribute_values []$V_{i}$, publicKey $Q_{U}$) →σ function GenerateProof(attribute values []$V_{i}$, private key $S_{U}$) → proof EV 01: // Step (i): Compute T based on the public parameters p and q 02: T=p⋅q 03: // Step (ii): Input attribute values $V_{i}$ and private key $S_{U}$ 04: // Compute the formula for F 05: F = empty set // Initialize F as an empty set 06: for each attribute value $v_{i_{j}}$ in $V_{i}$: 07:   // Compute $l_{j}$ 08:   $l_{j}=H_{2}\left(S_{U}\right)\left(v_{i_{j}}\right)$ 09:   // Square each $l_{j}$ to get $f_{j}$ 10:   $f_{j}=l_{j}^{2}$ 11:   // Add $f_{j}$ to the set F 12:   $F=F\;union\;\left\{f_{j}\right\}$ 13: // Construct the proof EV 14: $EV=\left\{T,\;F\right\}$ 15: return EV endfunction

Based on the above process, the CA and AA complete the registration of all end members $u_{i}$ and establish a registry of end member information for easy querying of member details. The specific contents are shown in Table 4.

(3) $Encrypt\left(M,W_{DO}\right)\to \left\{CT\right\}$ Encryption Algorithm

This algorithm is executed by the DO, who is an end member. The algorithm takes a message M as input and the attribute corresponding weight values $W_{DO}$, and outputs the ciphertext CT. The specific process is as follows:

The DO first selects a random number δ from the group $G_{T}$ to be used as the symmetric encryption key. Let the symmetric encryption algorithm be denoted by E, the DO encrypts the message M to be shared by calculating $E_{\delta }\left(M\right)=E\left(\delta ,M\right)$, resulting in the symmetric encrypted ciphertext $E_{\delta }\left(M\right)$. The DO stores the ciphertext $E_{\delta }\left(M\right)$ in IPFS, obtaining the data storage address $Address\left(E_{\delta }\left(M\right)\right)$, and then encrypts the symmetric key δ. The specific encryption process is as follows:

  i. The weight corresponding to the attribute set is $W_{DO}=\left\{w_{DO_{1}},w_{DO_{2}},\ldots ,w_{DO_{r}}\right\}$, with the total weight denoted as $W=\overset{r}{\underset{j=1}{\sum }}w_{DO_{j}}$. Randomly select W+1 prime numbers $b_{0},b_{1},\ldots ,b_{W}$, which satisfy the relation $b_{1}b_{2}\ldots b_{t}>b_{0}b_{W-t+2}b_{W-t+3}\ldots b_{W}$. Let $B=\left(b_{0},b_{1},\ldots ,b_{W}\right)$, then choose a random number β in $GF\left(b_{0}\right)$, and compute the following formula:
  (6) $$C=\delta u^{\beta }$$
 ii. Set the attribute weight threshold value as t, where 0<t≤W. Let $Y=b_{1}b_{2}\ldots b_{t}$, and select an integer A such that $0\leq A\leq \left[\frac{Y}{b_{0}}\right]-1$, then compute the following formula:
  (7) $$\mu =\beta +Ab_{0}$$

Then, the output ciphertext is:

(8) $$CT=\left(A,B,C\right)$$

The DO utilizes the regulatory node to store the ciphertext CT on the blockchain, simultaneously providing a brief description of the ciphertext. This is then associated with the relevant attribute categories and the data storage address.

The process of the encryption algorithm is as follows (Algorithm 3):
**Algorithm 3:** $Encrypt(M,W_{DO})\to \{CT\}$// Params: message M, attribute weights $W_{DO}$ // Returns: ciphertext CT function EncryptMessageWithAttributes(char[] M, int[] $W_{DO}$) →CT 01: // Select a random number δ from $G_{T}$ to be used as the symmetric encryption key 02: δ = selectRandom($G_{T}$) 03: // Encrypt the message M with δ using symmetric encryption E 04: $E_{\delta }\left(M\right)$ = symmetricEncrypt(δ, M) 05: // Store the encrypted message $E_{\delta }\left(M\right)$ on IPFS and get the storage address 06: Address_$E_{\delta }\left(M\right)$ = IPFS.store($E_{\delta }\left(M\right)$) 07: // Step (i): Compute the sum of weights W and select W+1 prime numbers $b_{0}$ to $b_{W}$ 08: $W=\mathrm{sum}\left(W_{DO}\right)$ 09: B= selectPrimes(W+1) 10: // Select a random number β from $GF\left(b_{0}\right)$ 11: β= selectRandom($GF\left(b_{0}\right)$) 12: // Encrypt δ using the formula given 13: $C=\delta u^{\beta }$ 14: // Step (ii): Set the attribute weight threshold t and compute μ 15: Y= product(B,1,t) // Product of the first t primes in B 16: A= selectInteger(0, floor($\left[\frac{Y}{b_{0}}\right]-1$) 17: $\mu =\beta +Ab_{0}$ 18: // Construct the ciphertext CT 19: $CT=\left(A,B,C\right)$ 20: // Store the ciphertext CT on the blockchain using a regulatory node 21: blockchain.store(CT) 22: // Provide a description of the ciphertext, link it with the attribute category and data storage address 23: blockchain.associate(CT, “description”, “attribute category”, Address_$E_{\delta }\left(M\right)$) 24: return CT endfunction

(4) $Identity\left(EV\right)\to \left\{Address\left(E_{\delta }\left(M\right)\right)\right\}$ Authentication Algorithm

This algorithm is jointly executed by the DO and DV within the terminal member environment. The DV inputs their zero-knowledge proof $EV_{DV}$, and the output is the authentication result and the data storage address $Address\left(E_{\delta }\left(M\right)\right)$. The specific process is as follows:

When a DV wishes to access the data M, they need to obtain the access address of this data in the IPFS storage system. At this point, the DV needs to submit their zero-knowledge proof $EV_{DV}$ to the DO, proving their identity. Once verified, the DO then sends the data storage address $Address\left(E_{\delta }\left(M\right)\right)$ to the DV. The detailed process is as follows:

  i. Based on the zero-knowledge proof $EV_{DV}$’s parameter T, the DV generates a random parameter σ within the range $\left(0,T\right)$ and then calculates a based on σ. The calculation formula is as follows:
  (9) $$a\equiv \sigma^{2}\;mod\;T$$
 ii. The DO generates a random sequence E and initiates an identity verification challenge to the DV.
  (10) $$E=\left\{e_{1},e_{2},\ldots ,e_{r}\right\},e_{j}\in \left\{0,1\right\}$$
iii. Upon receiving the challenge, the DV calculates the response parameter Res using their private key $S_{U}$ and sends it to the DO. The specific formula for calculating Res is as follows:
  (11) $$Res=\sigma \overset{r}{\underset{j=1}{\prod }}l_{j}^{e_{j}}\;mod\;T$$
 iv. The DO verifies using the zero-knowledge proof $EV_{DV}$ and response parameters Res, with the calculation formula as follows:
  (12) $$\gamma =\overset{r}{\underset{j=1}{\prod }}EV\cdot f_{j}^{e_{j}}\;mod\;T$$

When the equation $Res^{2}\equiv a\gamma \;mod\;T$ holds true, the identity verification of the DV is successful. The DO sends the data storage address, $Address\left(E_{\delta }\left(M\right)\right)$, to the DV and calculates the attribute permission parameters based on the DV’s weight values. If the equation does not hold, all operations are terminated.

  v. Once the DV’s authentication is successful, the DO calculates the attribute permission parameters based on DVs weight values. The specific calculation formula is as follows:
  (13) $$r_{j}=\mu \;mod\;b_{j}\;(1<j\leq r^{\prime })$$
  (14) $$R_{i}=\left\{r_{1},r_{2},\ldots ,r_{j}\right\}$$

In the formula, $b_{j}$ represents the product of any distinct $w_{j}$ primes chosen from $b_{0},b_{1},\ldots ,b_{W}$. After calculating the attribute permission parameter $R_{i}$, it is sent to the DV along with the data storage address $Address\left(E_{\delta }\left(M\right)\right)$.

The process of the authentication algorithm is as follows (Algorithm 4):
**Algorithm 4:** $Identity\left(EV_{DV}\right)\to \left\{Address\left(E_{\delta }\left(M\right)\right)\right\}$// Params: Zero-knowledge proof $EV_{DV}$ // Returns: Authentication result and the data storage address Address($E_{\delta }\left(M\right)$) function Verify Access And Retrieve Data (proof $EV_{DV}$) → {Address_$E_{\delta }\left(M\right)$} 01: // Step (i) Generates a random parameter σ based on T from $EV_{DV}$ 02: σ = select Random From (T) 03: // Compute a using σ 04: $a\equiv \sigma^{2}\;mod\;T$ 05: // Step (ii) Generates a random sequence E and issues an identity challenge to the DV 06: $E=\left\{e_{1},e_{2},\ldots ,e_{r}\right\},e_{j}\in \left\{0,1\right\}$. 07: sendChallenge (challenge) 08: // Step (iii) Computes the response parameters Res using their private key $S_{U}$ 09: Res=σ 10: for j from 1 to r do: 11:  $Res=Res\cdot l_{j}^{e_{j}}mod\;T$
12: sendResponse (Res) // Send the response parameters Res to the DO 13: // Step (iv) Verifies the proof $EV_{DV}$ and the response parameter Res 14: γ=1 15: for j from 1 to r do: 16:  $\gamma =\gamma \cdot EV\cdot f_{j}^{e_{j}}mod\;T$ 17: // Verify the equation 18: if $Res^{2}\equiv a\gamma \;mod\;T$: 19:  authentication Result = true 20:  sendAddress ($Address\left(E_{\delta }\left(M\right)\right)$) // DO sends the data storage address $Address\left(E_{\delta }\left(M\right)\right)$ to DV 21: // Step (v) Calculate attribute permission parameters based on the DV’s weight values 22:  for j from 1 to $r^{\prime }$ do: 23:    $r_{j}=\mu \;mod\;b_{j}$
24:    $R_{i}=\left\{r_{1},r_{2},\ldots ,r_{j}\right\}$ 25:    send Permission Parameters ($R_{i}$, $Address\left(E_{\delta }\left(M\right)\right)$) // Send attribute permission parameters $R_{i}$ and $Address\left(E_{\delta }\left(M\right)\right)$ to DV 26:  else: 27:  authentication Result = false 28:    // Terminate any further action if the equation does not hold 29:    terminate () 30:  // Return the authentication result 31:  return {authentication Result} endfunction

(5) $Encrypt\left(M\right)\to \left\{CT\right\}$ Decryption Algorithm

This algorithm is performed by the DV among end members. It takes the ciphertext CT and the data storage address $Address\left(E_{\delta }\left(M\right)\right)$ as input, and outputs the plaintext M. The specific process is as follows:

Based on the brief description of encrypted information by the DO in the blockchain, the DV quickly locates the ciphertext CT of the desired data in the blockchain. Then, using the obtained attribute permission parameters $R_{i}$, the DV decrypts the ciphertext.

  i. The following formula is computed based on the attribute permission parameter:
  (15) $$\left\{\begin{array}{c} \begin{array}{c} x\equiv r_{1}\left(mod\;b_{1}\right)\\ x\equiv r_{2}\left(mod\;b_{2}\right) \end{array}\\ \ldots \\ x\equiv r_{j}\left(mod\;b_{j}\right) \end{array}\right.$$

Using the standard Chinese remainder theorem, a unique solution can be calculated:

(16) $$x=\mu =\left(\overset{j}{\underset{v=1}{\sum }}r_{v}\cdot y_{v}\cdot \frac{Y}{b_{v}}\right)mod\;b_{v}$$

where $y_{v}\cdot \frac{Y}{b_{v}}\;mod\;b_{v}=1$. After obtaining μ, the value of β can be solved as $\beta =\mu -Ab_{0}$.

 ii. Locate the symmetrically encrypted data $E_{\delta }\left(M\right)$ using the data storage address $Address\left(E_{\delta }\left(M\right)\right)$ and download it. Then, compute the following formula:
  (17) $$\delta =\frac{C}{u\cdot e{\left(g_{1},g_{1}\right)}^{\beta }}$$
  (18) $$M=D_{\delta }\left(E_{\delta },\delta \right)$$

Finally, the desired data M is obtained.

The process of the decryption algorithm is as follows (Algorithm 5):
**Algorithm 5:** Encrypt(M)→{CT}// Params: ciphertext CT, attribute permission parameters $R_{i}$, data storage address Address($E_{\delta }\left(M\right)$) // Returns: plaintext message M function DecryptMessage ($CT,R_{i},$ Address_$E_{\delta }\left(M\right)$) →M 01: // Retrieve encrypted data $E_{\delta }\left(M\right)$ using the storage address from IPFS 02: $E_{\delta }\left(M\right)=$ IPFS.retrieve (Address_$E_{\delta }\left(M\right)$) 03: // Step (i): Compute μ using the attribute permission parameters $R_{i}$ 04: μ= computeCRTSolution ($R_{i}$, CT.B) 05: // Reverse compute β from μ 06: $\beta =\mu -Ab_{0}$ 07: // Step (ii): Decrypt δ using C from CT and β 08: u= publicParameters.u // u is obtained from the system’s public parameters 09: $g_{1}=$ publicParameters.$g_{1}$ // $g_{1}$ is a generator of $G_{1}$ 10: $\delta =\frac{C}{u\cdot e{\left(g_{1},g_{1}\right)}^{\beta }}$ 11: // Decrypt the message M using symmetric decryption with δ 12: M= symmetricDecrypt ($E_{\delta }\left(M\right),\delta$) 13: return M endfunction // Helper function to compute μ using Chinese Remainder Theorem and attribute permission parameters $R_{i}$ function computeCRTSolution ($R_{i},B$,) → int 01: Y= product ($b_{1},b_{t}$) // Y is the product of $b_{1},b_{2}\ldots ,b_{t}$ 02: μ=0 03: // Compute the sum for μ using the CRT formula 04: for v=1 to $R_{i}$.length do 05:   $y_{v}$ = modInverse ($\frac{Y}{b_{v}},b_{v}$) // Compute the modular inverse 06:   $\mu =\left(\mu +\left(r_{v}\cdot y_{v}\cdot \frac{Y}{b_{v}}\right)\right)\mathrm{mod}Y$
07: return μ endfunction

### 4.4. Blockchain Network Creation and Smart Contract Definition

Considering the target application within the IIoT, where the information stored in the terminal devices is typically highly confidential, we have opted for Hyperledger Fabric, tailored for IioT scenarios, as the development platform. Within this framework, we have designed and deployed two types of smart contracts on the blockchain: static smart contracts and dynamic smart contracts. The static smart contracts primarily take charge of managing pertinent information within the system following the initiation of data sharing requests by members. This encompasses operations such as information querying, updating, and uploading. On the other hand, dynamic smart contracts are responsible for real-time monitoring of members’ operational behaviors and access privileges. This ensures that the data sharing process adheres to the predetermined rule policies, including the enactment and revocation of access permissions.

During node deployment, we positioned the CA node on an isolated host, disconnected from the external internet, thereby effectively shielding the system from unauthorized external access and enhancing the system’s security and trustworthiness. Concurrently, we deployed peer nodes on physical nodes with internet connectivity, ensuring not only the continuity and reliability of external services but also enabling the peer nodes to store smart contract codes and process data access requests. This setup fosters effective interaction between the external environment and the blockchain network. Additionally, we distributed orderer nodes across multiple physical nodes. Together with the other modules of Hyperledger Fabric, these nodes form a highly secure, reliable, and high-performance blockchain network. The core modules of Fabric are detailed in Table 5.

**Figure 4:**
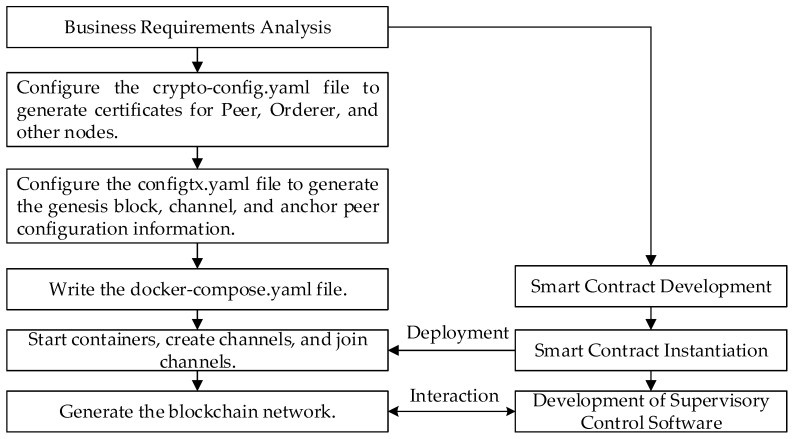
Blockchain network creation process.

The specific process of creating a blockchain network is shown in Figure 4.

## 5. Scheme Analysis

In this section, we provide a detailed analysis of the correctness and security of the scheme. Initially, we verify the correctness of the scheme from both theoretical and practical application standpoints, ensuring its stable functionality under various conditions. Subsequently, we explore the security of the scheme, focusing on its privacy protection performance and potential security threats.

### 5.1. Correctness Analysis

The correctness of this scheme can be demonstrated through the following theorem.

**Theorem** **1.** *Any legitimate end member* $u_{i}$ *in the system can prove their legitimacy and download the data resources they wish to access.*

**Proof of Theorem** **1.** In the registration phase of the scheme, each end member $u_{i}$ is provided with zero-knowledge proof evidence EV. When a DV applies to a DO for data access, the DO, based on the pre-reserved evidence EV from the zero-knowledge proof, generates a random sequence E and initiates a challenge. Upon receiving this challenge, the DV uses their private key $S_{DV}$ to generate a response parameter Res and replies accordingly. Since the DV’s private key $S_{DV}$ is kept secret, only the DV can generate the correct response parameter Res and provide valid proof of their identity.   □

Assuming the DV possesses an attribute set $A_{DV}=\left\{a_{DV1},a_{DV2},\ldots ,a_{DVr}\right\}$ with corresponding attribute values $V_{DV}=\left\{v_{DV1},v_{DV2},\ldots ,v_{DVr}\right\}$, then according to the non-interactive zero-knowledge proof protocol, the identity verification challenge posed by the DO expands from one bit to r bits for each session, assuming the challenge process is repeated for k rounds. In a one-bit challenge, the probability of the DV correctly guessing is 12. Therefore, at the end of the challenge, the probability of an impostor DV successfully deceiving the DO is only $Pr=2^{-kr}$. When kr is sufficiently large, Pr approaches zero.

Therefore, any legitimate end member $u_{i}$ in the system can prove their legitimacy based on the zero-knowledge proof pre-evidence and private key generated for them by the CA institution during the registration process. Once the identity of the DV is verified, the DO sends them the data storage address $Address\left(E_{\delta }\left(M\right)\right)$ and calculates the attribute permission parameters based on the weight values of the DV. The DV can then locate the ciphertext of the data resource they wish to access in the IPFS storage system using the storage address $Address\left(E_{\delta }\left(M\right)\right)$ and compute the decryption key based on the attribute permission parameters, ultimately obtaining access to the desired data resource.

**Theorem** **2.** *In the system, any end member* $u_{i}$ *whose sum of attribute permission values is greater than or equal to the threshold value set during encryption can calculate the decryption key and access the corresponding ciphertext resource.*

**Proof of Theorem** **2.** After the identity of the DV is verified, the DO calculates the attribute permission parameter R based on the DV’s weight values. The DV then uses the attribute permission parameter R to calculate the decryption key during the decryption phase. When the sum of the attribute permission values possessed by the DV reaches the threshold, $b_{1}b_{2}\ldots b_{j}\geq b_{1}b_{2}\ldots b_{t}$, the system of Equation (13) satisfies the congruence $r_{i}\equiv r_{j}mod\left(b_{i},b_{j}\right),i\neq j$. According to the generalized Chinese remainder theorem, Equation (11) has a unique solution x within the range $\left[0,b_{1}b_{2}\ldots b_{j}\right]$, which is μ. Thus, $\beta =\mu -Ab_{0}$ can be solved.   □

Therefore, in the system, any end member $u_{i}$ whose sum of attribute permission values is greater than or equal to the threshold value set during encryption can calculate the unique solution x, that is, μ, based on the attribute permission parameter R generated during the authentication phase, and subsequently solve for β. Then, using the ciphertext $C=\delta u^{\beta }$, the symmetric encryption/decryption key δ can be calculated as $\delta =\frac{C}{u.e{\left(g_{1},g_{1}\right)}^{\beta }}$. Following this, the desired data M can be computed using the decryption algorithm $D_{\delta }$.

### 5.2. Security Analysis

**Theorem** **3.** *Any legitimate end member* $u_{i}$ *in the system can anonymously access data resources without revealing their identity.*

**Proof of Theorem** **3.** During the registration phase, the CA institution generates zero-knowledge proof evidence EV based on the private key $S_{U}$ of the end member $u_{i}$. In the authentication phase, the DV only needs to present the evidence EV, allowing the DO to generate a random sequence $E=\left\{e_{1},e_{2},\ldots ,e_{r}\right\}$ based on the parameter T in the proof and initiate an identity verification challenge to the DV. Upon receiving the challenge, the DV merely needs to compute the response parameter Res using their private key $S_{U}$ and reply to the DO. The DO can then determine whether the DV possesses the corresponding private key $S_{DV}$ by checking if the equation $Res^{2}\equiv a\gamma \;mod\;T$ holds true based on the zero-knowledge proof evidence EV and the response parameter Res. Throughout this process, the DV does not need to disclose their real identity while still being able to verify their legitimacy. At the same time, impostor members without the correct private key cannot compute the correct response parameter Res. Since solving $Res^{2}\equiv a\gamma \;mod\;T$ is equivalent to factoring T→p.q, and large integer factorization is a known difficult problem, it is unlikely to be feasible.   □

Therefore, any legitimate end member $u_{i}$ in the system can securely prove the legitimacy of their identity without revealing their own identity, using the non-interactive zero-knowledge proof protocol. They can anonymously access the data resources in the system, ensuring the privacy of their identity with good anonymity.

**Theorem** **4.** *In the system, when the sum of the attribute permission values possessed by the DV is less than the threshold value set by the DO during encryption, the DV cannot access the data resources shared by the DO.*

**Proof of Theorem** **4.** After the identity of the DV is verified, the DO calculates the attribute permission parameter R based on the data visitor’s weight values. The DV then computes the decryption key during the decryption phase using the attribute permission parameter R they possess. When the sum of the attribute permission values held by the DV is less than the threshold value, $b_{1}b_{2}\ldots b_{j}<b_{1}b_{2}\ldots b_{t}$ holds. According to the generalized Chinese remainder theorem, the solution derived from Equation (16) is uniformly distributed across all congruence classes modulo $b_{0}$, meaning that although Equation (16) has solutions, they are not unique, and the solution x cannot be determined. Even if the congruence $r_{i}\equiv r_{j}mod\left(b_{i},b_{j}\right),i\neq j$ is satisfied, there isn’t enough information to determine the value of x, and no secret information can be obtained.   □

Therefore, in the system, any end member $u_{i}$ whose sum of attribute permission values is less than the threshold value set during encryption cannot calculate the unique solution x, that is, μ, based on the attribute permission parameter R generated during the authentication phase, and subsequently, they cannot solve for β. As a result, they also cannot compute the symmetric encryption/decryption key δ using the ciphertext C. Even if they obtain the data storage address $Address\left(E_{\delta }\left(M\right)\right)$, they cannot calculate the data M they wish to access.

## 6. Performance Analysis

In this section, we conduct a comprehensive evaluation of the proposed scheme’s performance, encompassing both theoretical and experimental aspects. The theoretical analysis primarily involves a comparative study of several relevant algorithms and a deep analysis of computational costs. Building on this, we designed and implemented a series of experiments to further validate the accuracy of the theoretical analysis and the practical performance of the scheme. This combination of theoretical and experimental approaches makes our analysis more comprehensive, ensuring the effectiveness and advancement of the scheme.

### 6.1. Theoretical Analysis

#### 6.1.1. Algorithm Characteristics Comparison

The data sharing scheme presented in this paper has been compared with the schemes in references [25,26,29,30,31] in terms of blockchain structure, identity authentication, privacy protection, combinational permission, and off-chain storage. The results of this comparison are shown in Table 6.

Through comparative analysis with the relevant literature, it has been observed that existing data sharing schemes for the Industrial Internet of Things (IIoT) still possess certain flaws.

Zhang A et al. designed a secure and privacy-preserving sharing mechanism for personal health information in electronic health systems, referred to as BSPP, based on blockchain technology. This scheme established two types of blockchains with specific data structures and consensus mechanisms: a private blockchain for storing personal health information and a consortium blockchain for managing secure indices of this information. However, this scheme lacks identity authentication and the capability for flexible combination of permissions. As a result, its applicability is quite limited and it falls short in terms of security.

Xue Y et al. proposed an attribute-based access control mechanism for data security and access control in public cloud storage environments, referred to as ABCCACS. This mechanism allows for more flexible and finer-grained access control policies, ensuring that only users with specific attributes or conditions can access the encrypted data stored in the cloud. However, the scheme does not verify the identity of users during data sharing, and only encrypts user information. Without authenticating the identities of end members in the system, the scheme cannot effectively prevent unauthorized access.

Zhang Q et al. proposed an access control scheme based on ciphertext-attribute authentication and threshold policies, referred to as AC-CAATP. This scheme employs hidden attribute-based identity authentication and divides permission levels through threshold functions set on user attributes. Users, based on the results of attribute verification, obtain varying levels of permissions, enabling access to data of different sensitivity levels on cloud servers. However, this scheme does not protect user information privacy during identity authentication, leading to the exposure of personal user information.

Liu H et al. proposed a lightweight non-interactive zero-knowledge proof protocol for verifying the existence of user private keys. This protocol allows the transmission of ciphertexts only after successful validation of the user’s private key. This effectively resolves the issue of high bandwidth usage in traditional CP-ABE systems, which is caused by unauthorized or invalid encryption data requests. However, in this scheme, attributes cannot be freely combined, and it involves directly storing encrypted data on the blockchain, increasing the storage burden.

Xu G et al. proposed a new medical data sharing scheme named PPMDS, which incorporates a blockchain-based authorization mechanism and attribute-based encryption algorithms to break system boundaries and enable data sharing among multiple medical institutions. However, this scheme lacks flexibility as it does not support the free combination of attributes in the process of data access control using attribute-based encryption.

Furthermore, the aforementioned schemes do not incorporate searchable capabilities, presenting limitations in retrieving and quickly locating specific data stored within the system. In response to the issues identified in existing data sharing schemes, our solution employs weighted threshold secret sharing technology to improve the data sharing mechanism, enhancing the flexibility and autonomy in permission configuration, making the data sharing process in the IIoT more adaptable. Additionally, the scheme utilizes non-interactive zero-knowledge proof protocols for preliminary authentication of data accessors, effectively preventing unauthorized user intrusion. Moreover, our solution adopts the IPFS distributed storage system to store encrypted resources, recording only storage addresses on the blockchain, significantly reducing the storage load. In summary, our solution addresses the deficiencies of existing schemes in several key areas, including blockchain architecture, identity authentication, privacy protection, flexible combination of permissions, off-chain storage, and fine-grained access control.

#### 6.1.2. Computational Cost Analysis

In data sharing schemes, the computationally intensive operations include hash function computations, bilinear pairing computations, and exponentiation operations, while the computational costs of simple addition and multiplication operations can be negligible. To understand the computational complexity of the aforementioned schemes more clearly, a comparison of computational costs in the key generation phase, authentication phase, encryption phase, and decryption phase between our scheme and five other schemes is made, providing a theoretical basis for further analyzing the computational overhead. Assuming that there are n end members participating in the data sharing process in the system, $T_{h}$ represents the computational cost of hash function operations, $T_{b}$ represents the computational cost of bilinear pairing operations, and $T_{e}$ represents the computational cost of exponentiation or modular exponentiation operations. $r_{s}$ represents the average number of attributes owned by end members and $r_{d}$ represents the average number of attributes used by DVs for decrypting ciphertext. The comparison of computational costs in the key generation phase, authentication phase, encryption phase, and decryption phase between our scheme and the other five schemes is shown in Table 7, where the key generation phase includes the initialization and registration stages of our scheme and the other schemes.

As indicated in Table 6, during the key generation phase the computational requirements of BSPP, ABCCACS, AC-CAATP, and ZK-CP-ABE are significantly greater than those of the proposed method. Furthermore, their computational demands increase linearly with the number of attributes held by end members. During the authentication phase, the computational demand of the proposed scheme is minimal when dealing with a small number of attribute values. As the attribute values increase, the computational requirements of this scheme show a linear growth and exceed that of other schemes. This is because the scheme employs a non-interactive zero-knowledge proof protocol. This protocol is used to generate zero-knowledge proofs of private keys for end members, thus ensuring their privacy is maintained throughout the authentication process. During the encryption and decryption phases, the proposed scheme requires the least computational resources when dealing with a small number of attribute values. As the number of attributes increases, the computational demand of this scheme becomes slightly higher than that of ZK-CP-ABE but remains lower than the other four methods. This is because the scheme, by characterizing and assigning values to attributes, achieves a flexible combination of permissions. This allows end members to access system data both flexibly and securely, a feature not realized by the ZK-CP-ABE approach.

### 6.2. Experimental Analysis

#### 6.2.1. Computational Analysis of Blockchain Operations

To evaluate the blockchain performance utilized in this study, we employed Caliper to test the write and read capabilities of the blockchain. The write operation refers to the DO writing data to the blockchain, which was conducted across four sets of tests. Each set simulated 1500 executions, varying the transaction submission rate. The submission speeds were set at 60 TPS (transactions per second), 90 TPS, 120 TPS, and 150 TPS, respectively. The read operation involves the DV reading data from the blockchain, repeating the procedure. The results of the blockchain system’s write and read throughput tests are presented in Table 8 and Table 9, respectively.

Based on the test results, the following conclusions are drawn:

(1) The blockchain is capable of processing transactions with a 100% success rate for both write and read operations.
(2) The throughput for write operations approximates the sending rate when it is below 90 TPS. When the sending rate exceeds 90 TPS, the throughput reaches its peak and latency increases sharply, indicating that the limiting sending rate for write operations is 90 TPS.
(3) The throughput for read operations approximates the sending rate when it is below 120 TPS. When the sending rate exceeds 120 TPS, the throughput peaks and latency increases sharply, suggesting that the limiting sending rate for read operations is 120 TPS.
(4) Overall, the average latency for write operations is significantly greater than for read operations, with write operations also exhibiting a higher average delay compared to read operations.

#### 6.2.2. Computational Analysis of the BBDSPP Scheme

**Figure 5:**
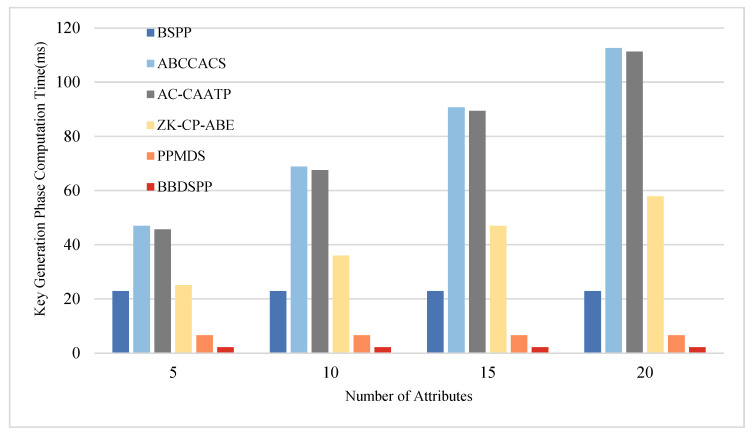
Comparative analysis of computational time in the key generation phase.

**Figure 6:**
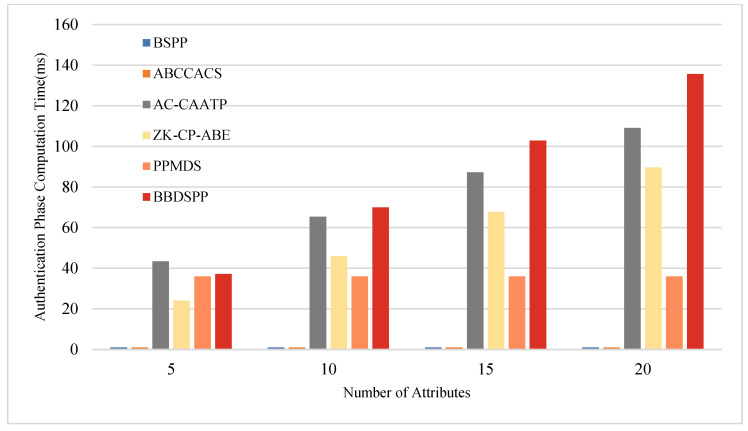
Comparative analysis of computational time in the authentication phase.

**Figure 7:**
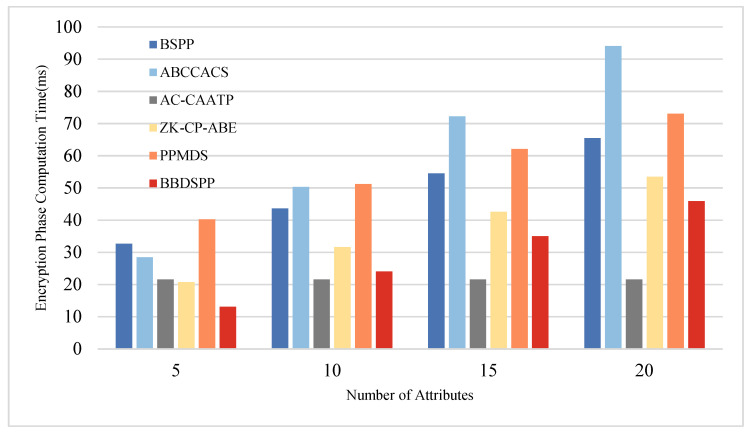
Comparative analysis of computational time in the encryption phase.

**Figure 8:**
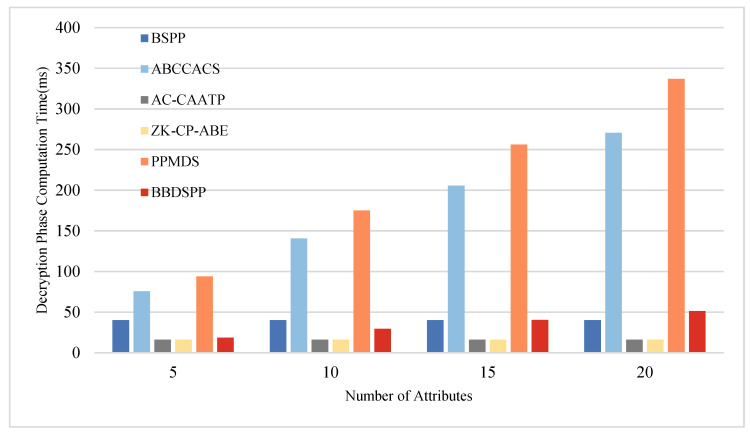
Comparative analysis of computational time in the decryption phase.

To further substantiate the theoretical analysis of each scheme, we conducted simulation tests using a laptop equipped with an i7 7500 u 3.0 GHz processor, 16 GB RAM, and 256 GB storage (Intel, Santa Clara, CA, USA), within a Python 3.12 software environment. To ensure the same level of security strength, the order p of the bilinear group $G_{1}$ is chosen as a 512-bit large prime number, and the order q of the bilinear group $G_{2}$ is selected as a 256-bit large prime number. Multiple operations are conducted, and the average values are taken as the final results. The average time for one hash function operation $T_{h}$ is approximately 0.00052 ms. The average time for one bilinear pairing operation $T_{b}$ is about 5.4005 ms, and the average time for one exponentiation or modular exponentiation operation $T_{e}$ is 2.1875 ms. Four different sets of attribute numbers $r_{s}$ possessed by end members are set, namely 5, 10, 15, and 20, to compare the time consumption in the four phases of key generation, authentication, encryption, and decryption. The specific time consumption comparison charts are shown in Figure 5, Figure 6, Figure 7 and Figure 8 and the specific values are detailed in Table 10, Table 11, Table 12 and Table 13.

During the key generation phase, the computational requirements of BSPP, ABCCACS, AC-CAATP, ZK-CP-ABE, and PPMDS significantly exceed those of our scheme. Moreover, as the number of attributes possessed by terminal members increases, their computational demands increase linearly. This indicates that in terms of key generation, our scheme has an advantage in efficiency, especially when the terminal members have a large number of attributes, resulting in lower computational overhead compared to other schemes.

During the authentication phase, AB-CCACS and ZK-CP-ABE lack an authentication process, resulting in a computational load of zero. When the number of attributes exceeds 10, the computational load of the scheme proposed in this paper is higher than that of the other four schemes. This is attributed to the fact that, in the authentication phase, our scheme generates a zero-knowledge proof for terminal members using attribute values, a process that leads to increased computational overhead with a larger number of attributes.

During the encryption phase, the computational load of the scheme presented in this paper is the lowest when the number of attributes is less than 10. However, when the number of attributes exceeds 10, the computational load of our scheme is only higher than that of AC-CAATP. This indicates that the scheme proposed in this paper is more efficient when dealing with a smaller number of attributes.

During the decryption phase, the computational load of the scheme proposed in this paper is slightly higher than that of AC-CAATP and ZK-CP-ABE. However, the increase in computational load is relatively small as the number of attributes possessed by the terminal members grows. This suggests that when dealing with a larger number of at-tributes, our scheme maintains a certain level of computational efficiency in processing decryption operations, being only marginally higher than certain other schemes.

**Figure 9:**
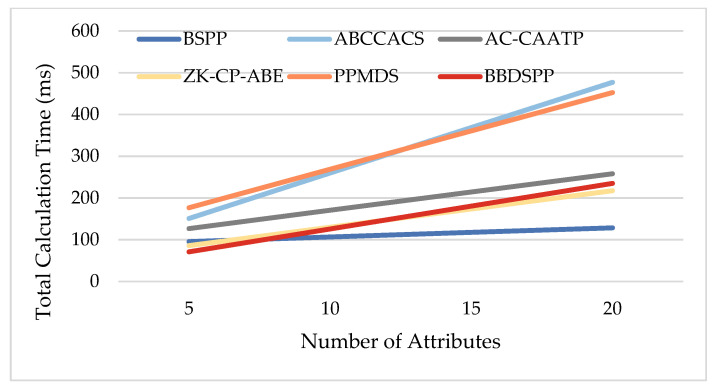
Comparative analysis of total calculation time for each scheme.

To facilitate a more direct and visual comparison of the computational costs of our scheme against others, we calculated the total time across all stages for attribute values of 5, 10, 15, and 20, and conducted a comparative analysis. The specific comparison of time consumption is depicted in the line chart in Figure 9, with detailed numerical values provided in Table 14.

From the above charts, it is evident that the total computational load of the BBDSPP scheme is consistently lower than that of ABCCACS, AC-CAATP, and PPMDS. It is slightly higher than ZK-CP-ABE when the attribute value exceeds 10. This is because our scheme employs a non-interactive zero-knowledge proof protocol to generate zero-knowledge proofs of private keys for terminal members and characterizes attribute values to realize flexible combination of permissions. This enables terminal members to access system data flexibly and securely. ZK-CP-ABE does not implement this feature. Since the BSPP lacks an identity authentication function, its total computation time is always lower than that of the BBDSPP scheme. In summary, by comparing the computational consumption of various schemes through simulation experiments, it is evident that the scheme proposed in this paper not only addresses the deficiencies of existing schemes but also demonstrates comparable or superior performance in terms of computational consumption, further validating the feasibility of our scheme.

## 7. Conclusions

In response to the issues of complex communication processes, poor flexibility, and low security in traditional data sharing models of the IIoT, we propose the BBDSPP scheme. Initially, we assign values to attributes based on their characteristics and utilize a weighted threshold secret sharing scheme to improve the data sharing process. This approach facilitates a data sharing access control mechanism that allows for the free combination of permissions. Terminal members can freely choose the attributes to decrypt. As long as the attribute values meet the preset access threshold, they can decrypt and access specific data. This not only ensures the flexibility of data sharing but also guarantees stringent control over data access.

Additionally, we employ non-interactive zero-knowledge proof protocols to pre-authenticate the identities of data accessors, preventing illegal members from impersonating and stealing sensitive data. This not only protects the privacy of terminal members but also avoids the extra computational burden caused by illegal access. It ensures that only legitimate and authorized terminal members can access data in the system, effectively preventing unauthorized access and leakage of sensitive information.

Furthermore, we utilize the IPFS distributed storage system to store encrypted shared resources. By storing a large amount of data on IPFS and only keeping corresponding storage addresses on the blockchain, we effectively resolve the storage efficiency issues prevalent in traditional blockchain applications. This approach not only ensures the security and integrity of data but also significantly enhances the efficiency of data retrieval and the scalability of the system.

Finally, we analyzed the correctness and security of the proposed solution, conducted a theoretical analysis of the solution’s functional characteristics and computational costs, and designed experiments for validation. The results show that the BBDSPP scheme can address the deficiencies in existing solutions in several key areas such as identity authentication, privacy protection, flexible combination of permissions, and off-chain storage, while also maintaining good performance and demonstrating strong feasibility.

However, the BBDSPP scheme still has some potential limitations and areas for improvement. For instance, scalability for large-scale IIoT systems and feasibility of practical deployment require further study. Additionally, performance metrics of the BBDSPP scheme in terms of privacy protection and data security need further optimization. Therefore, future work will focus on addressing these issues and further refining and improving the BBDSPP scheme.

## Figures and Tables

**Table 1:** Research achievements in data sharing.

Year	Author(s)	Scheme	Main Contributions	Deficiencies
2018	Liu et al. [24]	Blockchain-Based Efficient Data Collection and Sharing Scheme	The authors have created an efficient and secure system for data collection and sharing within the IIoT by integrating the Ethereum blockchain and Deep Reinforcement Learning (DRL).	Since the scheme is primarily designed for single-user scenarios, it may not be applicable in multi-user environments.
2019	Yingjie Xue et al. [25]	Attribute-Based Controlled Collaborative Access Scheme for Public Cloud Storage Management	The authors explored a unique access control scenario that enables multiple users with diverse attribute sets to acquire access permissions collaboratively. They proposed a new attribute-based access control scheme, which facilitates controlled collaborative access by designating transformation nodes within the access structure. This approach is intended to prevent collaborations not specified by the access policy and to avoid erroneous authorization of access requests.	Although the method proposed in the paper is innovative in terms of flexibility and fine-grained control, it still falls short in implementing collaborative access in multi-user environments.
2021	Han Liu et al. [26]	Ciphertext-Policy Attribute-Based Encryption for the Internet of Things Information Center Based on Zero-Knowledge Proof	The authors introduced a lightweight non-interactive zero-knowledge proof protocol to verify the existence of a user’s private key. This protocol permits ciphertext transmission only after successful validation of the user’s private key. This approach effectively addresses the issue of high bandwidth usage in traditional CP-ABE systems caused by unauthorized or invalid encryption data requests.	The protocol’s flexibility is suboptimal in scenarios where encryption policies or user attributes are subject to frequent changes.
2022	Li et al. [27]	Novel AGKA Protocol Based on Blockchain and IIoT Attributes	Based on the attribute information of IIoT devices, the authors utilize the decentralized, immutable, and secure characteristics of the blockchain to implement access control for protocol participants. The scheme employs the blockchain to store device attribute information and utilizes smart contracts to execute access policies and manage rules, ensuring that only devices with specific attributes participate in the protocol.	Although blockchains provide security protection in this scheme, additional mechanisms are still needed to ensure data privacy and security.
2022	Chin-Ling Chen et al. [28]	Blockchain-Based IIoT Enterprise Secure Data Transmission Scheme	The authors store encrypted IIoT data on the IPFS network and create a keyword index table on Hyperledger Fabric for data sharing. This scheme utilizes Fabric’s channels and custom chaincodes to achieve privacy protection and efficient data transmission, while employing elliptic curve digital signature algorithm (ECDSA) to ensure data integrity.	Although the scheme employs the ECDSA to ensure data security and integrity, it still has shortcomings when addressing security threats and privacy issues in large-scale distributed environments.
2022	Guangquan Xu et al. [29]	Blockchain and Attribute Encryption-Based Medical Data Sharing Scheme	The authors have proposed a blockchain-based scheme for medical data sharing, designed to address privacy breaches and system isolation issues prevalent in traditional medical systems. This scheme employs a blockchain-based authorization mechanism along with attribute-based encryption (ABE) technology to facilitate data sharing across various medical institutions, breaking the barriers of system boundaries. Moreover, it leverages ABE for scalable access control, enhancing the framework’s overall security and efficiency.	Although the scheme employs advanced encryption and authorization mechanisms, further exploration is needed for effectively managing and maintaining these mechanisms in real-world medical environments to ensure data security and privacy protection.

**Table 2:** Research achievements in the field of privacy protection.

Year	Author(s)	Scheme	Main Contributions	Deficiencies
2018	Aiqing Zhang et al. [30]	Blockchain Secure Privacy Protection Information Sharing Mechanism (BSPP)	The authors have proposed a scheme for securing and preserving the privacy of data sharing in electronic health systems. This scheme includes two types of blockchains: a private blockchain for storing personal health information and a consortium blockchain for managing secure indices of this information. The scheme employs public key encryption algorithms to encrypt personal health data, keywords, and identity information, ensuring data security, access control, and privacy protection.	In the paper, the authors did not conduct an exhaustive evaluation of the proposed scheme’s performance, leaving its feasibility in practical applications undetermined.
2019	Qikun Zhang et al. [31]	Access Control Scheme Based on Encrypted Attribute Authentication and Threshold Policy	The authors have proposed an IIoT access control scheme that is based on ciphertext-attribute authentication and threshold policies. In this scheme, identity information has been encrypted and stored on the blockchain, where it is verified through smart contracts and decentralized consensus algorithms. Furthermore, the scheme has utilized the anonymity and encryption capabilities of blockchain technology to protect personal information privacy during user authentication.	Although the authors utilized attribute-based encryption techniques in this scheme, they did not provide a detailed threat model or security analysis, nor did they thoroughly explore issues related to privacy protection.
2020	Li et al. [32]	Permissioned Blockchain-Based Anonymous and Traceable Aggregate Signature (PBATAS) Scheme	The authors have designed a blockchain signature scheme suitable for the IIoT. This scheme compresses signatures from different senders through aggregated signatures to save bandwidth, while maintaining the autonomous management capabilities of the IIoT. The scheme employs smart contracts to verify anonymous sources and shares encrypted information among entities.	Due to the use of aggregated signature technology in this scheme, the computational overhead is significant, and the practicality of the scheme remains to be enhanced.
2020	Qi et al. [33]	Cpds: Efficient and Privacy-Preserving Compressed Private Data Sharing Scheme	The authors have proposed a compressed private data sharing (CPDS) framework for efficiently and securely managing product data in the IIoT on the blockchain. This framework employs two novel mechanisms for storing compressed product data and for policy enforcement, enabling multiple industrial participants to share product data efficiently in a distributed environment while achieving fine-grained access control.	Due to the limited storage space on the blockchain, this scheme exhibits deficiencies in storage performance when handling large volumes of data.
2022	Deebak et al. [34]	Blockchain-Based Remote Mutual Authentication (B-RMA) Method	The authors utilize the decentralized, immutable, and secure features of blockchain, based on the attribute information of IIoT devices, to implement access control for protocol participants. The scheme involves storing device attribute information on the blockchain and employing smart contracts to execute access policies and management rules, ensuring that only devices with specific attributes participate in the protocol.	Although the blockchain provides security protection in this scheme, additional mechanisms are still required to ensure the privacy and security of data.
2022	Yue Wang et al. [35]	Blockchain-Based IIoT Privacy Information Secure Sharing Scheme	The authors have proposed a blockchain-based scheme for the secure sharing of private information. This scheme has initially abstracted smart factories as edge nodes and has established a decentralized, distributed, and trusted blockchain network on simulated edge devices using the Ethereum client. Furthermore, the scheme has introduced an intelligent ECDSA to ensure the ownership of information shared among edge nodes and has designed an incentive mechanism based on information attributes to encourage sharing among these nodes.	Although the authors have abstracted smart factories as edge nodes and built a decentralized blockchain network based on the Ethereum client, the actual deployment of this scheme in IIoT may face more complex technical and operational challenges.

**Table 3:** Notations and Their Meanings Used in the Scheme.

Notation	Description	Notation	Description
U	End-Member Set	EV	Zero-Knowledge Proof
ID	End-Member Identity Identifier Set	δ	Symmetric Encryption Key
A	Ordered Attribute Set	E	Symmetric Encryption Algorithm
V	Attribute Value Set	Address	Data Storage Address
W	Attribute Weight Set	R	Attribute Permission Parameter Set
$Q_{U}$	End-Member Public Key	M	Plaintext
pp	System Public Parameters	CT	Ciphertext

**Table 4:** End member registration information table.

Name	1st End Member	2nd End Member	...	nth End Member
End Member	$u_{1}$	$u_{2}$	…	$u_{n}$
Identity Identifier	$id_{1}$	$id_{2}$	…	$id_{n}$
Attribute Set	$A_{1}$	$A_{2}$	…	$A_{n}$
Attribute Value	$V_{1}$	$V_{2}$	…	$V_{n}$
Attribute Weight	$W_{1}$	$W_{2}$	…	$W_{n}$
Public Key	$Q_{u_{1}}$	$Q_{u_{2}}$	…	$Q_{u_{n}}$
Zero-Knowledge Proof	$EV_{1}$	$EV_{2}$	…	$EV_{n}$

**Table 5:** Core modules of fabric.

Module Name	Function
Peer	Maintains and stores the distributed ledger within the Fabric network
Orderer	Manages transaction ordering and consensus mechanisms
Configtxgen	Generates initial and update configuration files for the network
Configtxlator	Processes and translates network configuration files

**Table 6:** Comparison of characteristics of various data sharing schemes.

Author	Year	Scheme	1	2	3	4	5	6	7
Zhang A et al. [30]	2018	BSPP	√	×	√	×	√	√	√
Xue Y et al. [25]	2019	AB-CCACS	×	×	×	√	√	√	×
Zhang Q et al. [31]	2019	AC-CAATP	√	√	×	√	√	√	√
Liu H et al. [26]	2020	ZK-CP-ABE	√	√	√	×	×	√	×
Xu G et al. [29]	2023	PPMDS	√	√	√	×	×	×	√
Ours	2023	BBDSPP	√	√	√	√	√	√	√

Notes: (1) blockchain architecture, (2) identity authentication, (3) privacy protection, (4) combinational permission, (5) off-chain storage, (6) fine-grained access control, and (7) searchable. √ = Yes, × = no.

**Table 7:** Comparison of computational costs between our scheme and four others.

Phase	BSPP	ABCCACS	AC-CAATP	ZK-CP-ABE	PPMDS	BBDSPP
Key Generation	$8T_{e}+T_{b}+6T_{h}$	$\left(8+n+2r_{s}\right)T_{e}+T_{b}$	$\begin{array}{l} \left(1+2r_{s}\right)nT_{e}+\\ 4T_{b} \end{array}$	$\begin{array}{l} \left(4+r_{s}\right)T_{e}+\\ T_{b}+T_{h} \end{array}$	$3T_{e}$	$T_{e}+T_{h}$
Authentication	/	/	$2r_{s}T_{e}+4T_{b}$	$\left(1+2r_{s}\right)T_{e}$	$\begin{array}{l} 14T_{e}+\\ T_{b}+T_{h} \end{array}$	$\begin{array}{l} \left(2+3r_{s}\right)T_{e}+\\ T_{h} \end{array}$
Encryption	$\begin{array}{l} \left(5+r_{s}\right)T_{e}+\\ 2T_{b}+2T_{h} \end{array}$	$\left(3+2r_{d}\right)T_{e}$	$4T_{b}$	$\begin{array}{l} \left(2+r_{d}\right)T_{e}+\\ T_{b}+T_{h} \end{array}$	$\begin{array}{l} \left(6+r_{s}\right)T_{e}+\\ 3T_{b} \end{array}$	$\left(1+r_{s}\right)T_{e}$
Decryption	$6T_{e}+5T_{b}+12T_{h}$	$\begin{array}{l} r_{d}T_{e}+\\ \left(2+2r_{d}\right)T_{b} \end{array}$	$3T_{b}$	$3T_{b}$	$\begin{array}{l} T_{e}+\\ \left(2+3r_{d}\right)T_{b} \end{array}$	$\begin{array}{l} \left(1+r_{d}\right)T_{e}+\\ T_{b} \end{array}$

**Table 8:** Results of the write operation performance tests in the blockchain system.

Sending Speed (TPS)	Success	Failure	Maximum Latency (s)	Minimum Latency (s)	Average Latency (s)	Throughput (TPS)
60	1500	0	2.32	0.68	1.12	59
90	1500	0	3.65	1.23	1.45	87
120	1500	0	6.78	2.56	3.15	90
150	1500	0	8.64	3.14	3.85	91

**Table 9:** Results of the read operation performance tests in the blockchain system.

Sending Speed (TPS)	Success	Failure	Maximum Latency (s)	Minimum Latency (s)	Average Latency (s)	Throughput (TPS)
60	1500	0	0.78	0.04	0.24	60
90	1500	0	0.89	0.03	0.32	90
120	1500	0	1.23	0.05	0.38	117
150	1500	0	5.64	0.34	1.45	118

**Table 10:** Calculation time result of key generation stage.

	BSPP	ABCCACS	AC-CAATP	ZK-CP-ABE	PPMDS	BBDSPP
5	22.9036	46.9630	45.6645	25.0885	6.5625	2.1880
10	22.9036	68.8380	67.5395	36.0260	6.5625	2.1880
15	22.9036	90.7130	89.4145	46.9635	6.5625	2.1880
20	22.9036	112.5880	111.2895	57.9010	6.5625	2.1880

**Table 11:** Calculation time result of authentication phase.

	BSPP	ABCCACS	AC-CAATP	ZK-CP-ABE	PPMDS	BBDSPP
5	0	0	43.4770	24.0625	36.0260	37.1880
10	0	0	65.3520	45.9375	36.0260	70.0005
15	0	0	87.2270	67.8125	36.0260	102.8130
20	0	0	109.1020	89.6875	36.0260	135.6255

**Table 12:** Calculation time result of encryption phase.

	BSPP	ABCCACS	AC-CAATP	ZK-CP-ABE	PPMDS	BBDSPP
5	32.6770	28.4375	21.6020	20.7135	40.2640	13.1250
10	43.6145	50.3125	21.6020	31.6510	51.2015	24.0625
15	54.5520	72.1875	21.6020	42.5885	62.1390	35.0000
20	65.4895	94.0625	21.6020	53.5260	73.0765	45.9375

**Table 13:** Calculation time result of decryption phase.

	BSPP	ABCCACS	AC-CAATP	ZK-CP-ABE	PPMDS	BBDSPP
5	40.1337	75.7435	16.2015	16.2015	93.9960	18.5255
10	40.1337	140.6860	16.2015	16.2015	175.0035	29.4630
15	40.1337	205.6285	16.2015	16.2015	256.0110	40.4005
20	40.1337	270.5710	16.2015	16.2015	337.0185	51.3380

**Table 14:** Calculation time result of total calculation time for each scheme.

	BSPP	ABCCACS	AC-CAATP	ZK-CP-ABE	PPMDS	BBDSPP
5	95.7143	151.1440	126.9450	86.0660	176.8485	71.0265
10	106.6518	259.8365	170.6950	129.8160	268.7935	125.714
15	117.5893	368.5290	214.4450	173.5660	360.7385	180.4015
20	128.5268	477.2215	258.1950	217.3160	452.6835	235.0890

## Data Availability

All data are available from the corresponding author upon request.
